# Plant Tandem CCCH Zinc Finger Proteins Interact with ABA, Drought, and Stress Response Regulators in Processing-Bodies and Stress Granules

**DOI:** 10.1371/journal.pone.0151574

**Published:** 2016-03-15

**Authors:** Srimathi Bogamuwa, Jyan-Chyun Jang

**Affiliations:** 1 Department of Horticulture and Crop Science, The Ohio State University, Columbus, Ohio, United States of America; 2 Department of Molecular Genetics, The Ohio State University, Columbus, Ohio, United States of America; 3 Center for Applied Plant Sciences, The Ohio State University, Columbus, Ohio, United States of America; 4 Ohio Agriculture Research and Development Center, The Ohio State University, The Ohio State University, Columbus, Ohio, United States of America; Institute of Botany, Chinese Academy of Sciences, CHINA

## Abstract

Although multiple lines of evidence have indicated that *Arabidopsis thaliana* Tandem CCCH Zinc Finger proteins, AtTZF4, 5 and 6 are involved in ABA, GA and phytochrome mediated seed germination responses, the interacting proteins involved in these processes are unknown. Using yeast two-hybrid screens, we have identified 35 putative AtTZF5 interacting protein partners. Among them, Mediator of ABA-Regulated Dormancy 1 (MARD1) is highly expressed in seeds and involved in ABA signal transduction, while Responsive to Dehydration 21A (RD21A) is a well-documented stress responsive protein. Co-immunoprecipitation (Co-IP) and bimolecular fluorescence complementation (BiFC) assays were used to confirm that AtTZF5 can interact with MARD1 and RD21A in plant cells, and the interaction is mediated through TZF motif. In addition, AtTZF4 and 6 could also interact with MARD1 and RD21A in Y-2-H and BiFC assay, respectively. The protein-protein interactions apparently take place in processing bodies (PBs) and stress granules (SGs), because AtTZF5, MARD1 and RD21A could interact and co-localize with each other and they all can co-localize with the same PB and SG markers in plant cells.

## Introduction

In living organisms, protein machineries govern almost all cellular processes in response to internal and external cues. It is rare that proteins act alone, instead, they interact with each other to orchestrate biological functions, such as transcriptional control of gene expression [1], cellular signal transduction [2], intracellular protein trafficking [3], cell cycle progression [4–6] and various types of cellular metabolism [7].

*Arabidopsis thaliana* tandem CCCH zinc finger proteins (AtTZFs) are potent regulators of plant growth and stress responses. AtTZF1 can shuttle between nucleus and cytoplasmic foci and co-localize with processing body (P-body or PB) and stress granule (SG) markers [8]. PBs and SGs are aggregations of cytoplasmic messenger RiboNucleoProtein (mRNP) complexes, playing important roles in post-transcriptional regulation and epigenetic modulation of gene expression [9]. Whereas PBs are enriched with translation-repressed mRNAs and mRNA silencing and degradation machineries [10, 11], SGs act as mRNA storage sites mainly containing stalled pre-initiation complexes and translation regulators [12, 13]. AtTZF1 can affect plant growth and development, enhance stress tolerance, modulate ABA, GA and sugar responsive gene expression and act as a positive regulator for ABA and a negative regulator for GA responses [14]. Notably, AtTZF1 can bind both DNA and RNA *in vitro* [8]. Recently, it has been found that the plant-unique arginine-rich (RR) and TZF motif (RR-TZF) of AtTZF1 is required for RNA binding [15]. In contrast to a more ubiquitous temporal and spatial expression pattern of *AtTZF1*, *AtTZF4*, *5* and *6* are specifically expressed in seeds. AtTZF4, 5, and 6 act as positive regulators for ABA and negative regulators for GA and phytochrome mediated seed germination responses by mediating ABA and GA biosynthetic and response gene expression. Like AtTZF1, AtTZF4, 5, and 6 can also co-localize with PB and SG markers in plant cells [16].

Human tristetraprolin (hTTP), a prototype of TZF proteins, can trigger rapid decay of ARE-containing mRNAs encoding cytokines and interleukins [17, 18]. TTP plays a pivotal role in PB/SG mediated mRNA metabolism in mammals, given TTP can nucleate PBs by recruiting and activating decapping and 5’-to-3’ mRNA decay machineries to degrade targeted mRNAs [19–22]. Results of co-immunoprecipitation assays show that hTTP is present in the same complex with decapping enzyme 1a (hDCP1a), decapping enzyme 2 (hDCP2), a deadeynylase called Carbon Catabolite Repressor 4 (hCCR4), and 5’-to-3’ exoribonuclease 1 (hXRN1) [19, 20]. In addition, a component of human exosome, hRRP4, could be co-purified with hTTP, indicating that hTTP might also be involved in 3’-to-5’ exonuleolytic decay [19]. It was identified that N-terminal domain of hTTP was involved in the interaction with mRNA decay enzymes [19]. Paradoxically, a recent report showed that a region in the C-terminal domain of hTTP could directly interact with central domain of CNOT1, a core subunit of CCR4- Negative on TATA (CCR4-NOT) complex [23]. This C-terminal domain is conserved in hTTP homologs, including Butyrate Response Factor 1 (BRF-1) and BRF-2 [23]. Using yeast two-hybrid screens, an additional 31 potential hTTP interacting partners were identified [24]. The interaction between hTTP and one of the putative protein partners, CBL-interacting protein 85 (CIN85) was mediated through a proline-arginine rich motif in the C-terminus of hTTP [24].

Other than N- and C-termini, hTTP also interacts with other proteins via its central TZF domain. For example, an isoform of AUF1 (an AU-rich element binding protein), AUFp45 and its related protein laAUF1, can interact with hTTP via TZF motif [25]. TTP is also involved in the inhibition of poly(A)-tail synthesis of ARE-containing nuclear mRNAs [26]. TTP could directly interact with poly(A)-binding protein nuclear 1 (PABPN1) via TZF motif of TTP and PABPN1 C-terminal RNA Recognize Motif (RRM) and Arginine-rich region (RR) [26]. Moreover, PABPN1 also interacts with poly(A) polymerase (PAP) via RR region. As TTP also interacts with PAP via TZF motif and it can bind PAP and PABPN1 simultaneously, it has been hypothesized that TTP may compete with the interaction between PABPN1 and PAP [26].

Compared with animals, plant species have a larger number of TZF genes due to genome-wide segmental duplication and tandem duplication [27–30]. For example, humans have three TZF genes [18] whereas Arabidopsis and rice have 11 and 9, respectively [30]. Although almost all members have been genetically characterized in *Arabidopsis thaliana* [31], functions of RR-TZFs in other plant species are not well studied. To date, only two reports have described RR-TZF protein-protein interactions in plants. Cotton (*Gossypium hirsutum*) Zinc Finger Protein 1 (GhZFP1) has been shown to interact with Responsive to Dehydration protein 21A (GZIRD21A) and Pathogenesis-Related protein 5 (GZIPR5) [32]. The interactions were mainly mediated through both TZF motif and the N-terminal region. A positive region at the C-terminus and a negative region at the N-terminus for protein-protein interaction were also identified [32]. *Arabidopsis thaliana* AtTZF9 is a phospho-target of mitogen-activated protein kinase 3 and 6 (MPK3 and MPK6). AtTZF9 directly interacts with MPK3 and MPK6 in cytosol and nucleus *in vivo*. Reverse genetic analyses indicate that MPK-targeted AtTZF9 is involved in pathogen associated molecular pattern (PAMP)- triggered immunity (PTI) [33].

In this study, we have attempted to identify AtTZF4, 5, and 6 interacting partners. Results showed that AtTZF4, 5, and 6 did not interact with conserved candidate partners such as decapping complex components and poly(A) binding proteins. Using Y-2-H library screens, 35 putative AtTZF5 interacting proteins were identified. Among them, Mediator of ABA-Regulated Dormancy 1 (MARD1) and Responsive to Dehydration 21A (RD21A) were chosen for further characterization. Results of bimolecular fluorescence complementation (BiFC) and co-immunoprecipitation (co-IP) analyses confirmed that AtTZF5 could interact with MARD1 and RD21A, respectively, in plant cells. The interaction with MARD1 and RD21A appeared to be mediated by TZF motif of AtTZF5. Finally, the interactions between AtTZF5 and MARD1/RD21A were likely taken place in PBs and SGs.

## Materials and Methods

### Plant materials and growth conditions

*Arabidopsis thaliana* and *Nicotiana benthamiana* were grown on SUNSHINE, LC1 Professional Growing Mix (Sun Gro Horticulture Distribution Inc., Agawam, MA, USA) in growth chambers at 22°C and 25°C, respectively, under a 16-h light and 8-h dark photoperiod. Arabidopsis and maize protoplast isolation and transformation were carried out as described [8, 34, 35].

### Molecular cloning

Unless stated otherwise, all cDNA clones were obtained from Arabidopsis Biological Resource Center (ABRC, Columbus, OH). Full-length coding sequences of *AtTZF1* (At2g25900), *AtTZF4* (At1g03790), *AtTZF5* (At5g44260), *AtTZF6* (At5g07500), and *AtTZF5 TZF* and *RR-TZF* motifs were cloned into pAS1 Y-2-H bait vector with ADH promoter and HA-tagged Gal4 DNA binding domain [36]. Full-length coding sequences of *MARD1* (At3g63210) and *RD21A* (At1g47128) were cloned into pAD-GAL4-2.1 prey vector containing GAL4 activation domain (Stratagene, Santa Clara, CA, USA). Oligo-primers used for cloning of coding sequences are listed in S1 Table.

For Bimolecular Fluorescence Complementation (BiFC) assay, coding sequences of *AtTZF4*, *AtTZF5*, *AtTZF6*, *AtTZF5*’s *TZF* motif, *AtTZF5*’s *RR-TZF* motif, *AtMARD1* and *AtRD21A* without stop codons were cloned into *CaMV35S-pA7-CYFP* and *CaMV35S-pA7-NYFP* vectors [37], respectively. Oligo-primers used for cloning are listed in S1 Table.

Molecular cloning for co-immunoprecipitation assay was carried out using Gatway^™^ (Invitrogen, Carlsbad, CA, USA) technologies according to manufacturer’s instructions. *AtTZF5* full-length coding sequence, *TZF5-RR-TZF* and *TZF5-TZF* were obtained by PCR using cDNA clone (U09506) obtained from ABRC (Columbus, OH, USA), and inserted into pENTR/D-TOPO^™^ and transferred to pK7FWG2 vector by Gateway LR recombination reactions. Oligo-primers used for cloning are listed in S1 Table. *AtMARD1* (At3g63210) full-length coding sequence was transferred into pGWB15 binary vector via Gateway LR recombination by using the cDNA clone (U16956) obtained from ABRC. The AtMARD1 was fused with HA tag in pGWB15 vector. For sub-cellular localization and co-localization analyses, full-length cDNA of *AtMARD1* (At3g63210) *AtRD21A* (At1g47128), *AtDIN10* (At5g20250), *AtPP2A-4* (At3g58500) and *Oleosin* (At5g56100) were transferred to a GFP tagged destination vector by Gateway LR recombination. This destination vector was modified from pBluescript KS+, in which the multi-cloning sites were replaced by a recombination cassette derived from pK7FWG2.0 [8]. *AtTZF5* full-length cDNA was cloned into a mCherry tagged destination vector (http://www.pnas.org/content/suppl/2007/03/26/0701061104.DC1) by Gateway LR recombination reaction.

### Yeast two-hybrid screens

PJ69-4A yeast strain was used for Y-2-H assays. Candidate interaction analysis was performed by co-expression of the respective bait and prey. Yeast transformation and Y-2-H analysis were performed as described [38]. Two Arabidopsis cDNA libraries were used for Y-2-H screens: CD4-22 made from 3-day-old etiolated Col-0 seedlings and CD4-30 made from inflorescence meristem, floral meristem and floral buds up to stage 8 or 9. Yeast plasmid DNA was extracted and transformed into *Escherichia coli* cells (DH5α strain). Plasmid DNAs were isolated from *E*. *coli* cells and the resulting sequences were used in blast search to identify corresponding cDNAs. To confirm the interactions identified from initial screens, individual cDNAs in lamda-ACT vector were re-transformed into PJ69-4A strain containing AtTZF5 in pAS1 bait vector and the growth assay was repeated.

### Imaging analysis

The BiFC analyses were conducted using a protoplast transient expression system [37]. Arabidopsis protoplast isolation and transformation were carried out as described [34, 35]. The paired BiFC constructs were co-transformed into protoplasts. A Nikon Eclipse E600 fluorescence microscope was used to document sub-cellular localization patterns of reporter fusion proteins. YFP fluorescence was visualized using an excitation filter (450 to 490 nm) and an emission filter (520 to 560 nm). Images of cells with positive YFP signals were taken by exposing under green channel. Whereas images of cells without YFP signals were taken using all three channels (red, green, and blue) to show cell integrities (red fluorescence from chloroplasts). Images were captured by using a SPOT RT Slider multimode camera and Advanced SPOT^™^ software (Diagnostics Instruments, Sterling Heights, MI, USA). For co-localization experiment, mCherry fluorescence was visualized using an excitation filter (555 to 560 nm) and an emission filter (630 to 660 nm). Co-localization analysis was conducted by merging GFP and mCherry images using SPOT Software 5.0 (Diagnostics Instruments, Sterling Heights, MI, USA).

### Co-immunoprecipitation assay

*AtTZF5* full-length, TZF and RR-TZF fragments in pK7FWG2 binary vector with GFP tag and *AtMARD1* full length cDNA in pGWB15 binary vector with HA tag were transformed into *Agrobacterium tumefaciens* strain GV3101 by electroporation. Resulting positive single colony was inoculated into 5 ml of LB and grown overnight at 28°C until OD_600_ reached 0.8. *Agrobacterium tumefaciens* strain harboring p19 was also grown similarly. The p19 is a 19 kDa viral protein that inhibits the onset of transgene-induced local and systemic silencing [39]. *Agrobacterium* cells were collected by centrifugation at 4,000 rpm for 10 minutes. Cells were then resuspended in half-volume (v/v) of freshly prepared infiltration buffer (10 mM MgCl_2_, 10 mM MES, 100 μM acetosyringone) and let stand for 2 h at room temperature. Cell suspension of AtMARD1 was then mixed with AtTZF5 full length or AtTZF5-TZF or AtTZF5-RR-TZF and p19 with the ratio of 1:1:2. Leaves of 3-wk-old *N*. *benthamiana* were infiltrated with *Agrobacterium* cell suspension using 1 ml needleless syringes. After infiltration, plants were grown in a growth chamber at 25°C under a 16 h light photoperiod for three days. The co-immunoprecipitation was performed as described [40]: 1) Agarose beads (20 μg, rProteinA Sepharose^™^ Fast Flow, GE Healthecare Bioscience Uppsala, Sweden) were washed with cold protein extraction buffer (200 mM Tris-HCl, pH 7.6, 400 mM NaCl, 20 mM EDTA, 4 mM DTT, 4% Lgepal CA-630, 4X complete protease inhibitor) for 4–5 times; 2) The beads were incubated with rabbit monoclonal anti-GFP antibody (Invitrogen-Molecular Probes, Eugene, Oregon, USA) for overnight at 4°C; 3) Beads were incubated with 500 μl protein extracts for 2 h at 4°C on a rotator (20 rpm); 4) Samples were centrifuged at 3,000 rpm at 4°C for 1min; 5) Beads were washed with 1 ml wash buffer #1 (WB1; 50 mM Tris-HCl pH 7.5, 120 mM NaCl, 1 mM EDTA, 1 mM PMSF, 1 mM DTT) by inverting gently for 5 times followed by centrifugation at 3,000 rpm at 4°C for 1 min; 6) Supernatant was removed and beads were washed with 1 ml WB2 (50 mM Tris-HCl pH 7.5, 120 mM NaCl, 1 mM EDTA, 1 mM DTT) twice and centrifuged at 3,000 rpm at 4°C for 1 min; 7) The beads were washed with 1 ml WB4 (1 ml of 1M Tris HCl, pH 8.0, 2 μl of 1M DTT) and centrifuged at 3,000 rpm at 4°C for 1 min. The supernatants were carefully removed to equalize the final volume of all the samples. Protein-loading buffer (50 mM Tris-HCl pH 6.8, 2% SDS, 10% glycerol, 1% β-mercaptoethanol, 12.5 mM EDTA, 0.02% bromophenol blue) was added and 20 μl of each sample was loaded onto SDS-PAGE gel for Western blot analysis.

### Western blot analysis

For all sodium dodecyl sulfate polyacrylamide gel electrophoresis (SDS-PAGE), 12.5% acrylamide gels were used. Plant protein isolation and gel-blot analysis were conducted as described [41]. For HA and GFP tagged proteins, mouse monoclonal anti-HA antibody (Roche Diagnostics, Indianapolis, IN, USA) and rabbit monoclonal anti-GFP antibody (Invitrogen-Molecular probes, Eugene, Oregon, USA) were used, respectively. The following secondary antibodies were used for immunoblotting: donkey anti-rabbit IgG, HRP conjugated secondary antibody (GE Healthcare UK Limited, Buckinghamsire, UK) and sheep anti-mouse IgG, HRP conjugated secondary antibody (GE Healthcare UK Limited, Buckinghamsire, UK).

## Results

### Identification of AtTZF5 interacting partners

**Fig 1:**
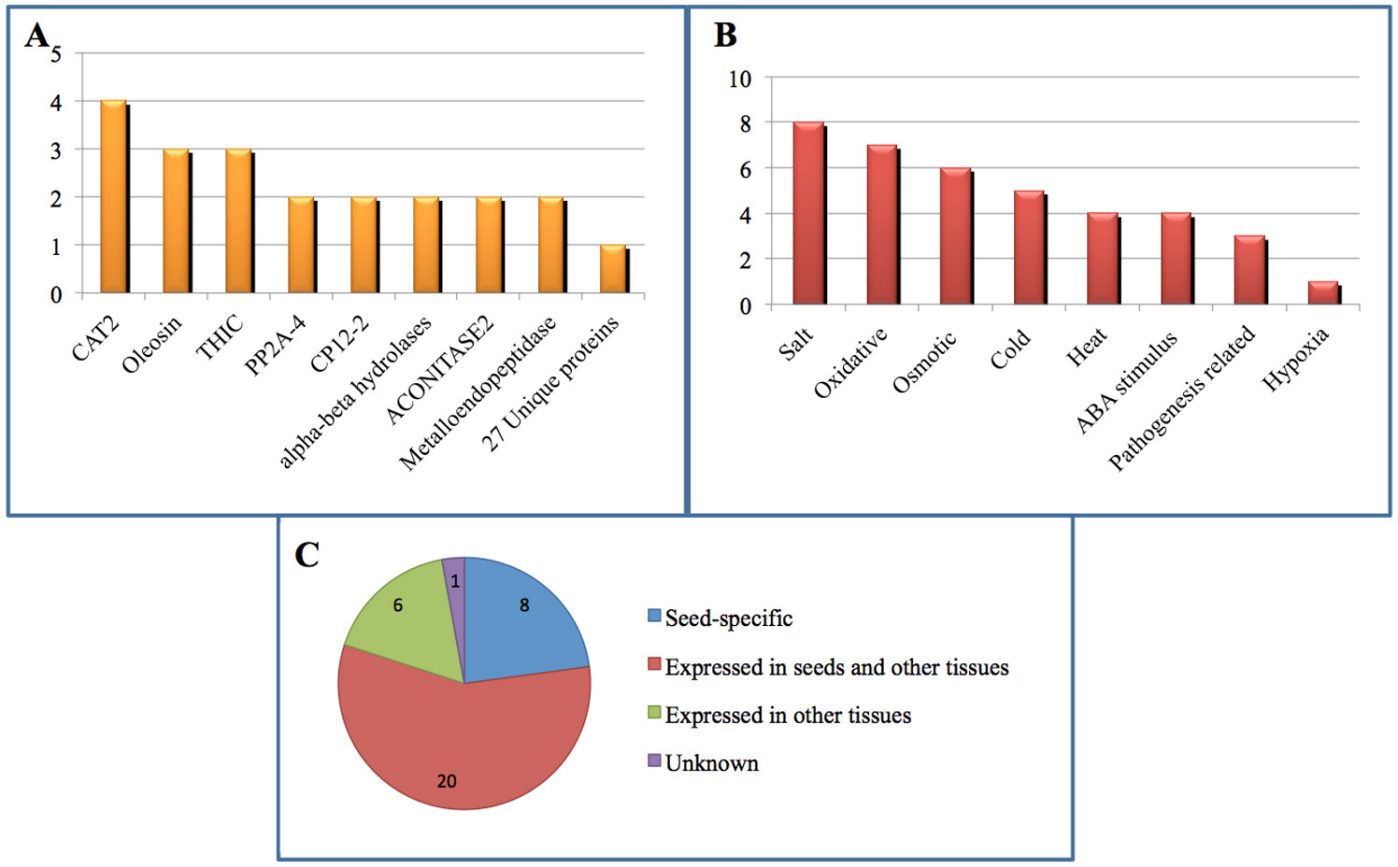
Putative AtTZF5 interacting partners identified by Y-2-H screens. (A) The representation of 35 proteins identified by Y-2-H screens. (B) Nineteen out of 35 proteins are involved in stress responses. (C) Tissue expression patterns of 35 identified protein-coding genes.

Y-2-H library screenings were conducted to identify interacting partners of AtTZF4, 5 and 6. Results of screening using AtTZF5 as a bait is reported here, because it produced the highest yeast transformation efficiency. Since seed specific libraries were unavailable, cDNA library from 3-day-old etiolated seedlings and inflorescence, respectively, were used for screening. Curiously, positive results were obtained only from 3-day-old etiolated seedlings library screening. Approximately 4x10^5^ colonies were screened and 47 positive clones corresponding to 35 different cDNAs were identified (S2 Table). Among them, CAT2 (Catalase 2), Oleosin, THIC (Pyrimidine Requiring Thiamin C), PP2A-4 (Protein Phosphatase 2A-4), CP12-2 (CP12 domain-containing protein), alpha-beta hydrolases, ACONITASE2, and Metalloendopeptidases were over-represented (Fig 1A). Results of Gene Ontology (GO) analysis (https://www.arabidopsis.org/servlets/Search?action=new_search&type=keyword and http://bar.utoronto.ca/efp/cgi-bin/efpWeb.cgi) indicated that 19 out of 35 candidates were involved in different types of stress responses including salt, oxidation, cold, heat, ABA, pathogens and hypoxia (Fig 1B and S3 Table). Since non-seed specific libraries were screened, the temporal and spatial expression patterns of all the identified genes were determined by using Arabidopsis eFP browser (http://bar.utoronto.ca/efp/cgi-bin/efpWeb.cgi). Interestingly, 28 of the genes were expressed in seeds; among them 8 were seed-specific (Fig 1C and S4 Table). Meanwhile, 6 of the genes were not expressed in seeds and one gene was not annotated (Fig 1C).

### Subcellular localization of AtTZF5 interacting partners

To determine if AtTZF5’s putative interacting partners could also localize to cytoplasmic foci, subcellular localization analysis was conducted by using a protoplast transient expression assay. The candidate proteins were fused with green fluorescent protein (GFP) as reporters. As shown in S1 Fig, MARD1 (Mediator of ABA Regulated Dormancy 1), RD21A (Response to Dehydration 21A), and PP2A-4 (Protein Phosphatase 2A-4) were localized to the cytoplasmic foci similar to AtTZF5. In contrast, DIN10 (Dark inducible 10) was localized to the nucleus. Meanwhile, Oleosin-GFP showed cytoplasmic diffusive localization that was similar to free GFP.

### AtTZF5 interacts with MARD1 and RD21A in plant cells

**Fig 2:**
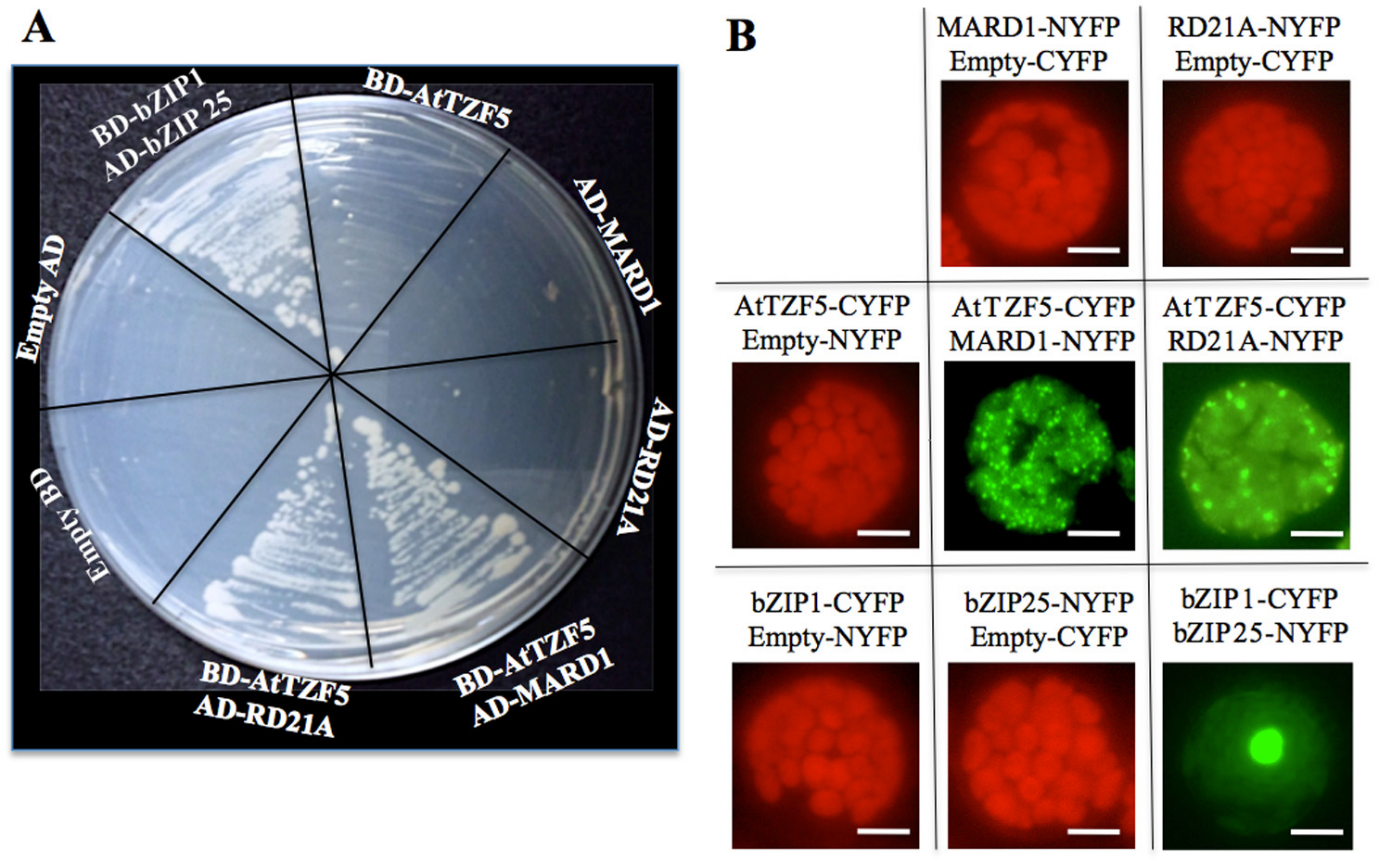
AtTZF5 interacts with MARD1 and RD21A. (A) AtTZF5 interacts with MARD1 and RD21A in a Y-2-H analysis. AtTZF5 was fused with GAL4 DNA binding domain (BD), whereas MARD1 and RD21A were fused with GAL4 activation domain (AD). The bZIP1+bZIP25 was used as a positive control pair. (B) Results of bimolecular fluorescence complementation (BiFC) analysis indicate that AtTZF5 interacts with MARD1 and RD21A in the cytoplasmic foci in Arabidopsis protoplasts. Interaction between bZIP1-CYFP and bZIP25-NYFP in the nucleus was used as a positive control. Images of cells with positive YFP signals were taken by exposing under green channel. Whereas images of cells without YFP signals were taken using all three channels (red, green, and blue) to show cell integrities (red fluorescence from chloroplasts). These experiments were repeated twice. Bar = 10μm.

**Fig 3:**
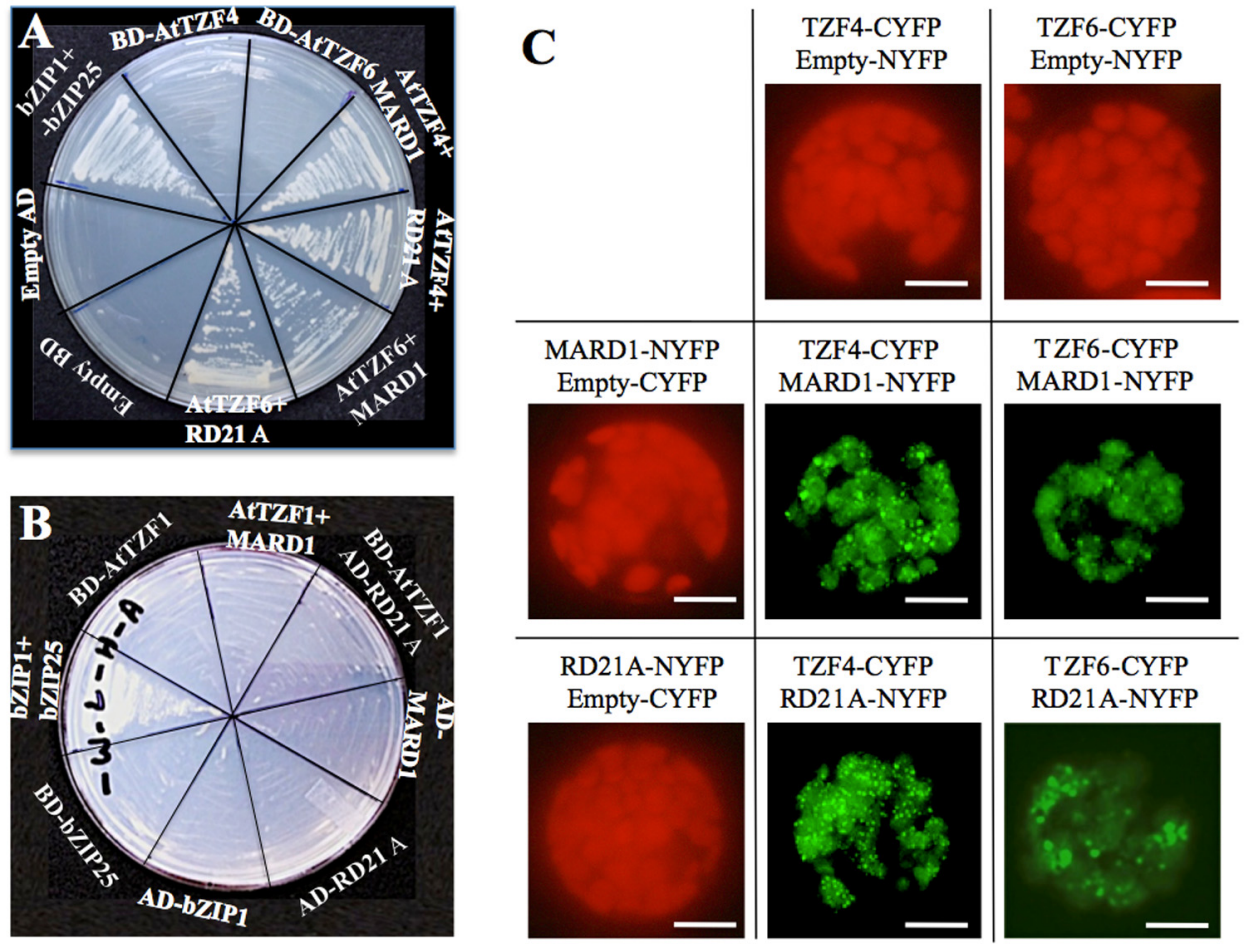
MARD1 and RD21A can interact with AtTZF4 and AtTZF6. (A, B) MARD1 and RD21A can interact with AtTZF4 and 6 but not AtTZF1 in Y-2-H analysis. AtTZF1, 4, and 6 were fused with GAL4 DNA binding domain (BD), whereas MARD1 and RD21A were fused with GAL4 activation domain (AD). (C) BiFC results indicate that MARD1 and RD21A can interact with AtTZF4 and AtTZF6 in cytolasmic foci in Arabidopsis protoplasts. Images of cells with positive YFP signals were taken by exposing under green channel. Whereas images of cells without YFP signals were taken using all three channels (red, green, and blue) to show cell integrities (red fluorescence from chloroplasts). These experiments were repeated twice. Bar = 10μm.

**Fig 4:**
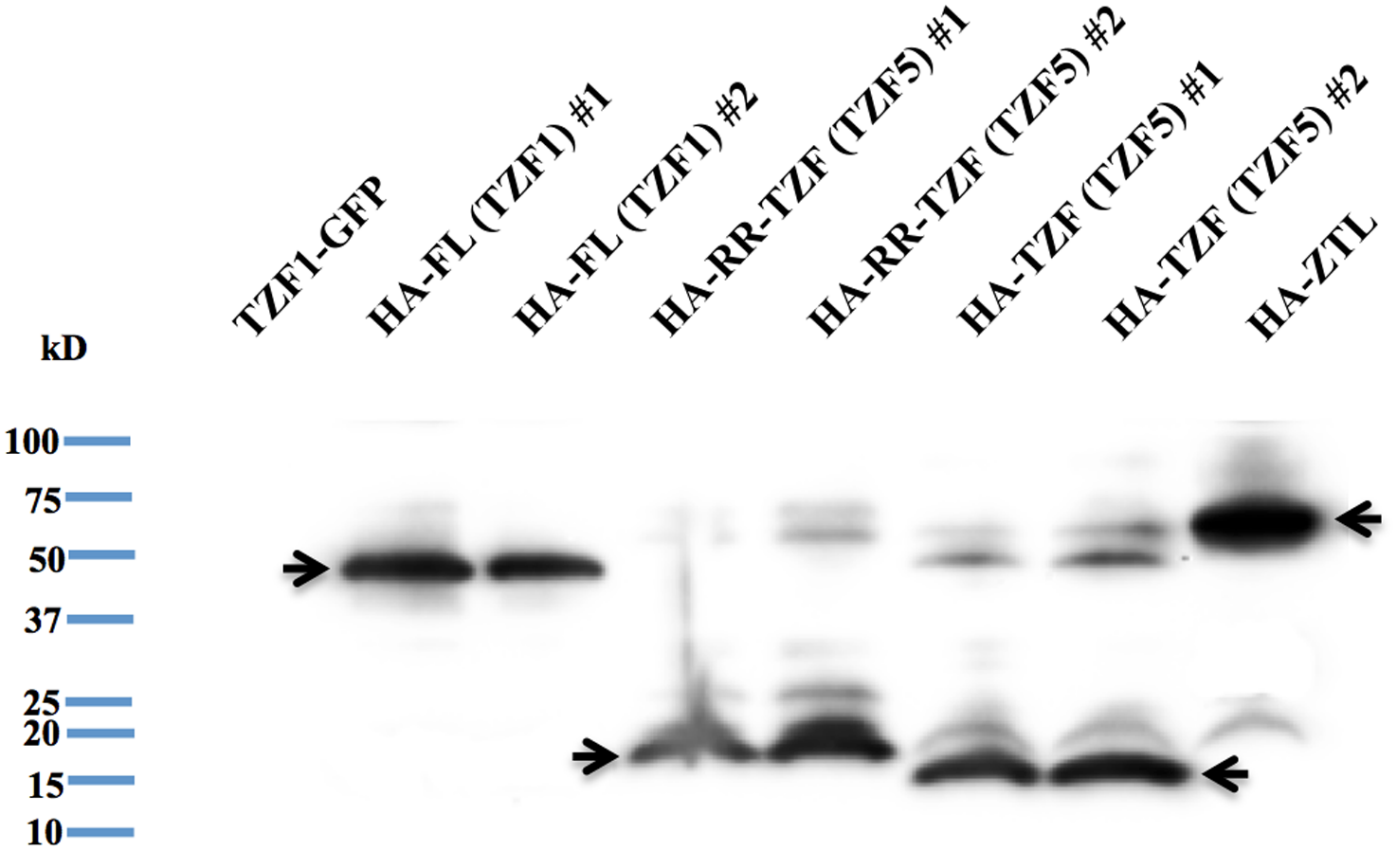
AtTZF1 (FL), RR-TZF and TZF of AtTZF5 were expressed in duplicate yeast cell lines. Shown are results of Western blot analyses. Full-length AtTZF1 as well as RR-TZF and TZF of AtTZF5 were fused with GAL4 DNA binding domain in HA tagged pAS1 vector. HA-ZTL was used as a positive control for the expression in yeast cells.

MARD1 (At3g63210) and RD21A (At1g47128) were chosen for further characterization because MARD1 is seed-specific and involved in ABA signal transduction [42], whereas cotton RD21A (GZIRD21A) also interacts with a cotton TZF protein GhZFP1 in Y-2-H analysis [32]. Using Y-2-H analysis, it was confirmed that full-length AtTZF5 interacted with full-length MARD1 and RD21A, respectively (Fig 2A). Bimolecular fluorescence complementation (BiFC) analysis was then used to confirm the interactions in plant cells. Yellow fluorescent protein (YFP) was detected in cytoplasmic foci in protoplasts transfected with AtTZF5-CYFP and MARD1-NYFP and RD21A-NYFP, respectively (Fig 2B). By contrast, no YFP signals were detected in protoplasts expression AtTZF1-CYFP, MARD1-NYFP, or RD21A-NYFP with its matching empty vector, indicating that AtTZF5 interacts with MARD1 and RD21A in cytoplasmic foci in plant cells. Y-2-H analysis was also conducted to determine if MARD1 and RD21A could interact with AtTZF1, AtTZF4 and AtTZF6. Results indicated that AtTZF4 and AtTZF6, but not AtTZF1, could also interact with MARD1 and RD21A, respectively, in yeast cells (Fig 3A). Consistent results were obtained from BiFC analyses (Fig 3C). Results of Western blot analysis indicated that negative interaction between AtTZF1 and MARD1 or RD21A was not due to the lack of expression in yeast cells (Fig 4).

Because mammalian TZFs (e.g., TTP) could interact with both PB [19] and SG [25, 26] components, attempts were also made to determine if some of the protein-protein interactions were conserved between mammalian TZFs and AtTZFs. However, results of Y-2-H analysis showed that AtTZF4, 5, and 6 could interact with neither PB (decapping complex components including DCP1, 2, and 5) nor SG (polyA-binding protein PABP8) marker proteins (S2 Fig).

### Delineation of AtTZF5 protein-protein interaction domains

**Fig 5:**
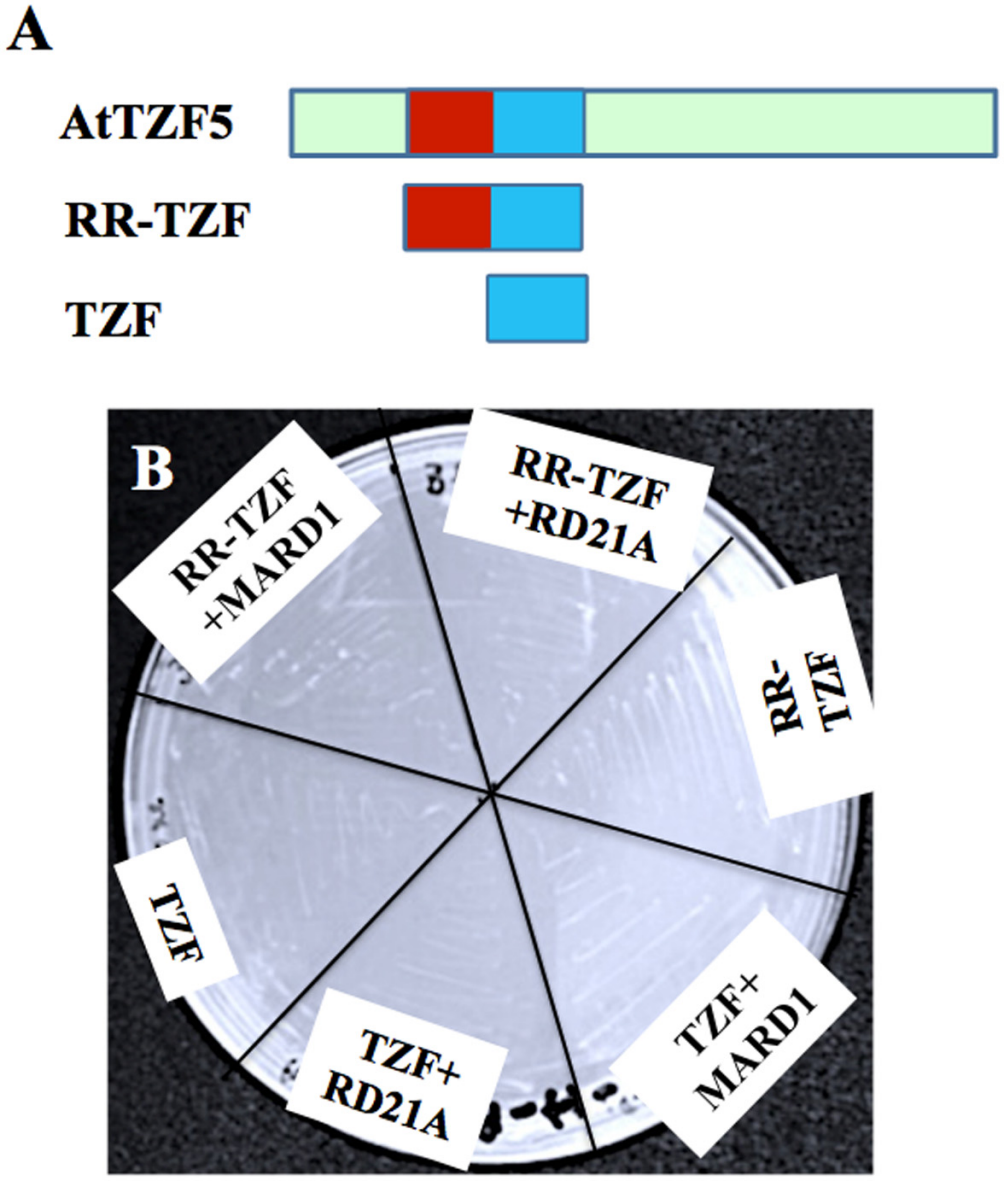
TZF and RR-TZF fragments of AtTZF5 cannot interact with MARD1 and RD21A in Y-2-H analysis. (A) AtTZF5 fragments used in this analysis. (B) Yeast cells fail to grow due to negative interaction between AtTZF5 truncated fragments (TZF and RR-TZF) and MARD1/RD21A. TZF, and RR-TZF of AtTZF5 were fused with GAL4 DNA binding domain (BD), whereas MARD1 and RD21A were fused with GAL4 activation domain (AD). This experiment was repeated three times.

**Fig 6:**
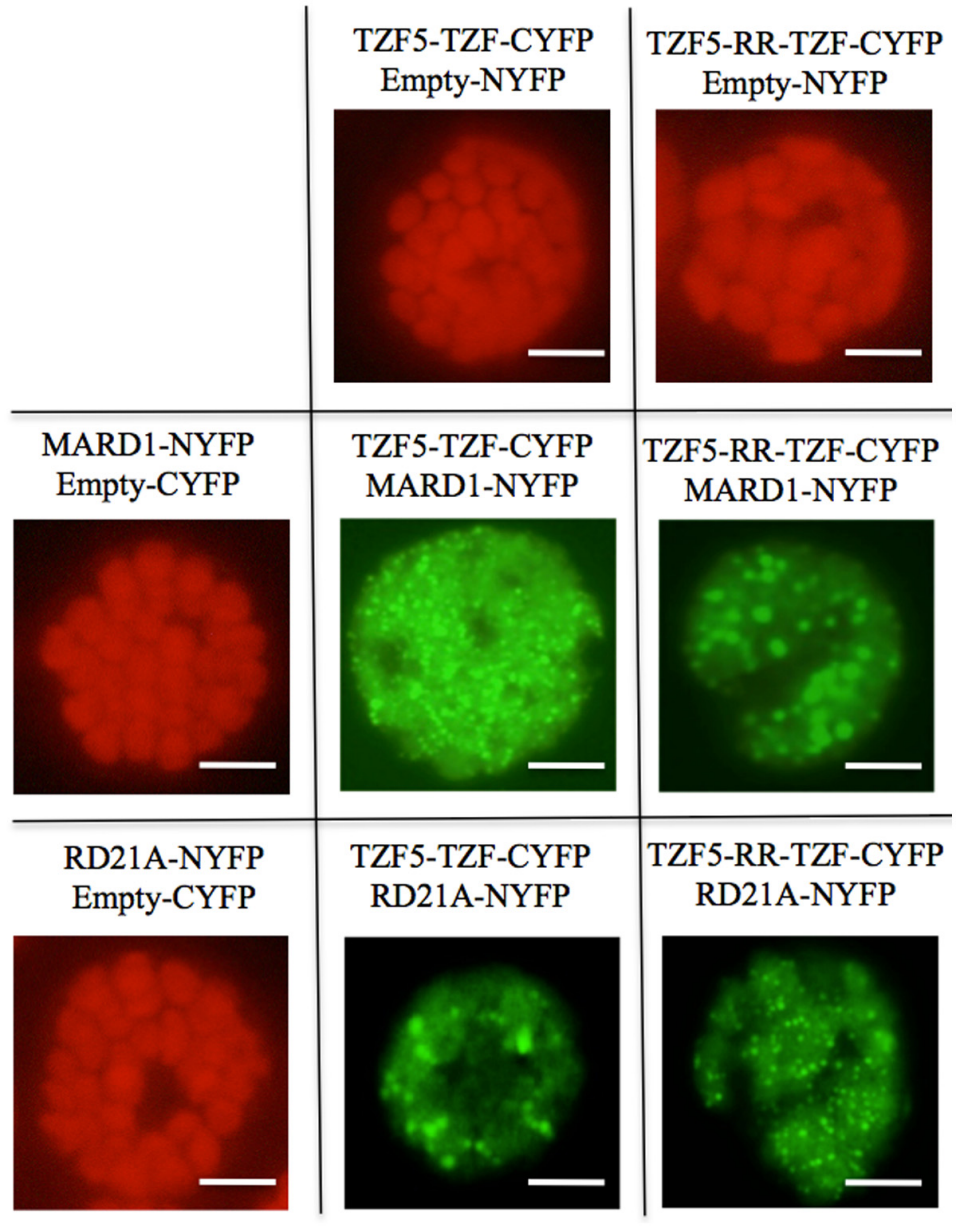
TZF and RR-TZF fragments of AtTZF5 can interact with MARD1 or RD21A in cytoplasmic foci. Shown are results of BiFC analysis using an Arabidopsis protoplast transient expression system. Images of cells with positive YFP signals were taken by exposing under green channel. Whereas images of cells without YFP signals were taken using all three channels (red, green, and blue) to show cell integrities (red fluorescence from chloroplasts). This experiment was repeated twice. Bar = 10μm.

**Fig 7:**
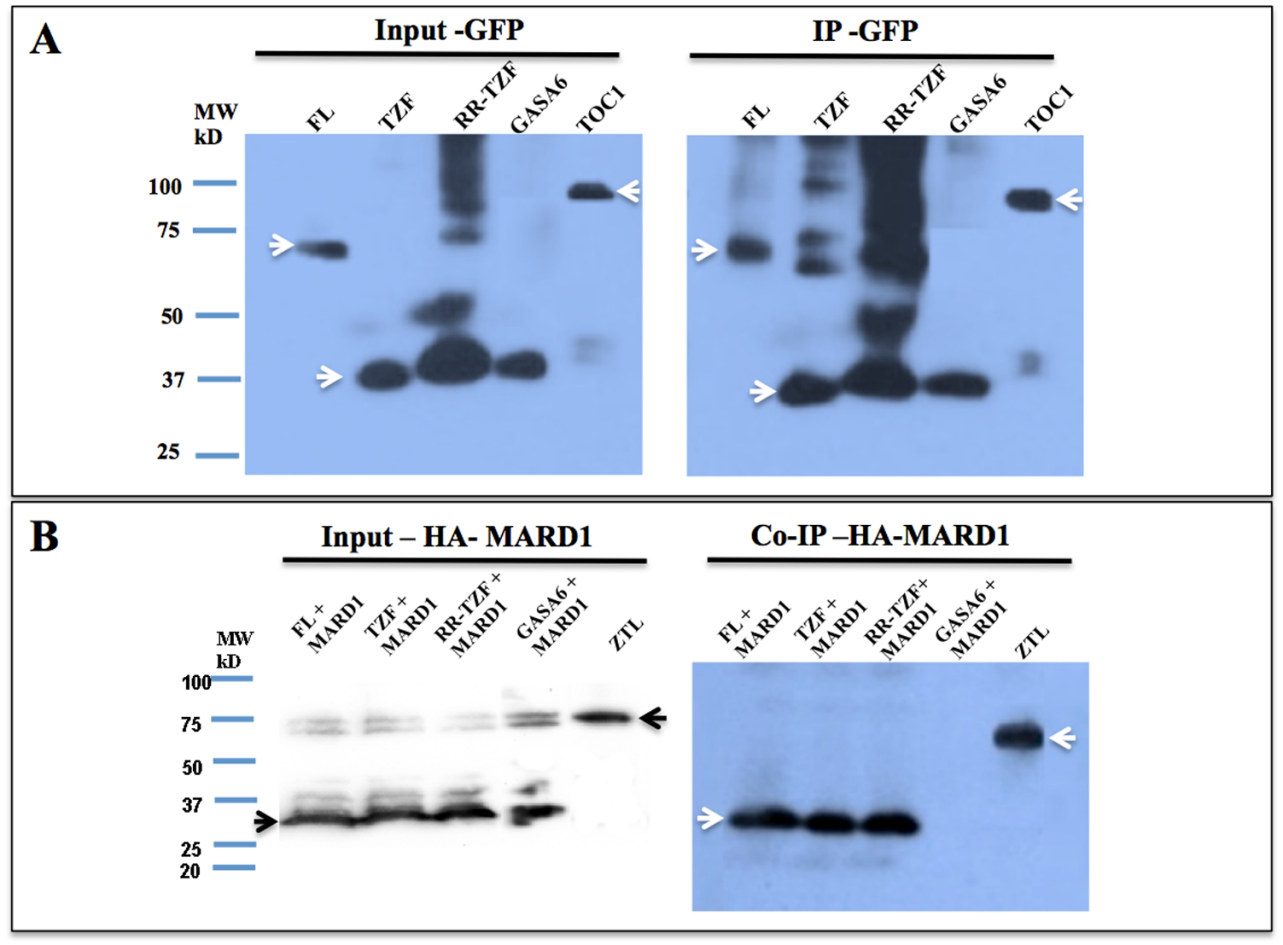
TZF domain of AtTZF5 is sufficient for interaction with MARD1 in co-immunoprecipitation analysis. AtTZF5 FL, TZF and RR-TZF fragments as well as GASA6 and TOC1 were tagged with GFP. MARD1 and ZTL were tagged with HA. GASA6 was used as a non-interacting control with MARD1. TOC1 and ZTL were used as a positive interacting pair. (A) Left panel shows signals of various input GFP tagged proteins (indicated by arrows). Anti-GFP antibody was used to pull down GFP-tagged proteins and revealed by Western blot analysis (arrow in right panel). (B) Left panel shows signals (indicated by arrows) of various input HA tagged proteins. Co-IP products were detected by anti-HA antibody as indicated by arrows in right panel.

To identify the region of AtTZF5 required for the interaction with MARD1 and RD21A, a deletion analysis was conducted. Because both RR and TZF regions of AtTZF1 are required for high affinity RNA binding [15], they were tested for protein-protein interaction (Fig 5A). In contrast to the full-length (FL) AtTZF5 (Fig 2A), neither TZF nor RR-TZF protein fragments could interact with MARD1 or RD21A in yeast cells (Fig 5B). Results of Western blot analysis indicated that both TZF and RR-TZF fragments were expressed normally in yeast cells (Fig 4). BiFC analysis was then conducted to confirm Y-2-H analysis results. Surprisingly, both TZF and RR-TZF protein fragments could interact with MARD1 and RD21A in cytoplasmic foci in Arabidopsis protoplasts, albeit only a few cells showed strong positive signals (Fig 6). Co-immunoprecipitation assay was then performed to further confirm the interaction between AtTZF5 and MARD1. Results showed that MARD1 interacted with AtTZF5 FL, TZF and RR-TZF protein fragments in co-immunoprecipitation assays (Fig 7). In contrast, negative control GA stimulated Arabidopsis 6 (GASA6) did not interact with MARD1 (Fig 7). Timing of CAB expression 1 (TOC1) was used as a positive control, which interacted with ZTL (ZTL-HA) [43] in the same experiment. Taken together, these results indicate that TZF region of AtTZF5 is sufficient to interact with MARD1 *in vivo*.

### AtTZF5 interacts with MARD1 and RD21A in cytoplasmic foci

**Fig 8:**
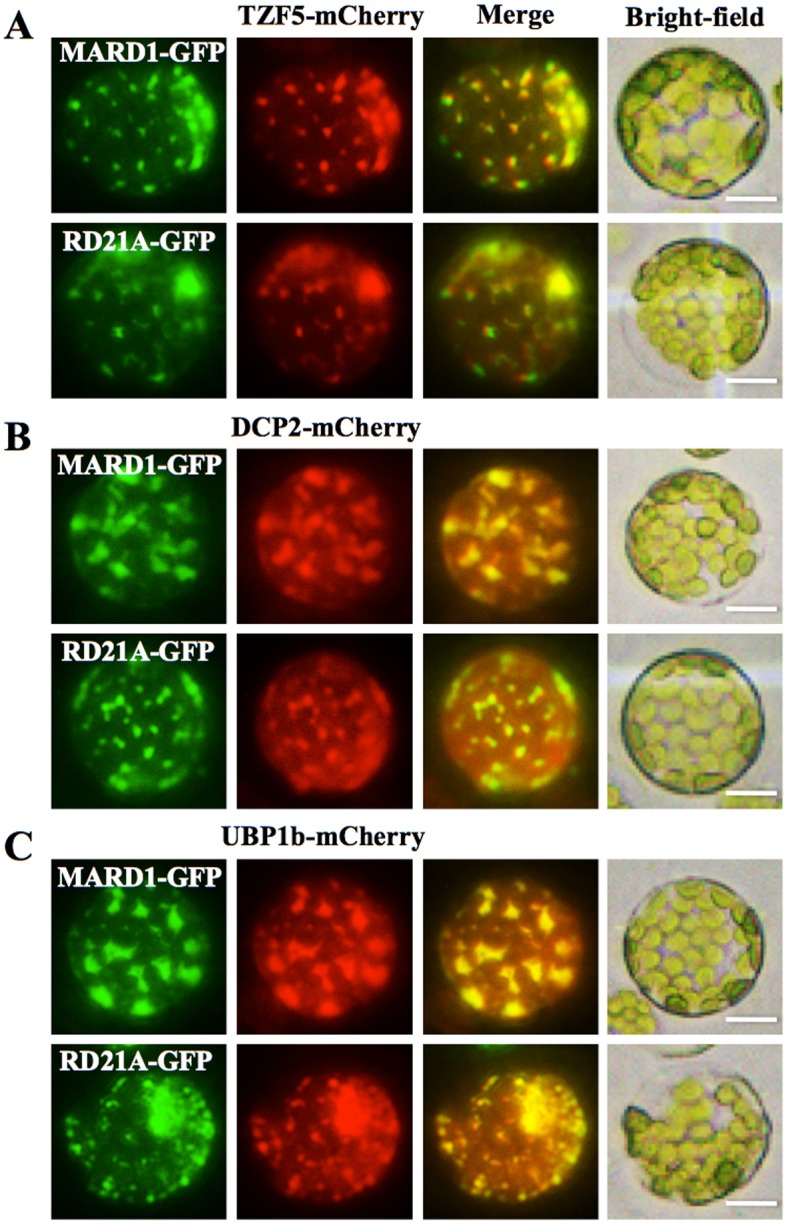
MARD1 and RD21A can co-localize with AtTZF5 and PB (DCP2) and SG (UBP1b) markers in protoplast transient expression analyses. (A) MARD1 and RD21A can co-localize with AtTZF5 in cytoplasmic foci. (B) MARD1 and RD21A can co-localize with PB marker DCP2. (C) MARD1 and RD21A can co-localize with SG marker UBP1b. Cellular images for GFP and mCherry signals were taken using green and red channel, respectively. Bright field images were also shown for cell integrities. Bar = 10μm.

To reveal the cellular locations where AtTZF5 interacted with MARD1 and RD21A, co-localization experiments were performed by using maize protoplast transient expression assays. As shown in Fig 8A, MARD1 and RD21A co-localized with AtTZF5 in the cytoplasm foci. Previously, we have shown that AtTZF5 can co-localize with both PB and SG markers [16]. Results of co-localization experiments showed that MARD1 and RD21A could also co-localize with PB marker DCP2 and SG marker UBP1b, respectively (Fig 8B and 8C). It was noted that the number and size of the co-localized cytoplasmic foci appeared to be variable, consistent with a general characteristic of PBs and SGs under different internal and external cues [8, 16, 44]. Taken together, these results suggest that AtTZF5 is likely to interact with MARD1 and RD21A in PBs and SGs.

## Discussion

Plant TZF proteins are potent regulators of growth, development and stress responses [31]. For example, *Arabidopsis thaliana* TZF1 (AtTZF1) affects plant growth and stress responses by mediating ABA-, GA- and sugar-responsive gene expression [14]. On the other hand, seed specific AtTZF4, 5 and 6 can act as positive regulators of ABA- and negative regulators of GA- and light-mediated seed germination responses [16]. AtTZF4, 5 and 6 affect ABA and GA biosynthetic and responsive gene expression during seed germination [16]. Because AtTZF1 can bind specific RNA elements and trigger mRNA degradation [15], and AtTZF1, 4, 5, and 6 can all co-localize with PB and SG markers [8, 16], it is tempting to speculate that protein-protein interaction is involved in AtTZF-mediated cellular processes and post-transcriptional regulation of gene expression. In this report, we have identified Mediator of ABA-Regulated Dormancy 1 (MARD1) and Responsive to Dehydration 21A (RD21A) as AtTZF4, 5, 6 interacting partners. Together with subcellular localization information, our results suggest that interacting protein partners may work in concert in PBs and SGs to control certain cellular activities, which in turn affecting seed dormancy and germination.

Given both hTTP [19, 25] and AtTZF1 [15] could bind mRNA and trigger mRNA degradation, mRNA deacy machineries were thought to be the top candidates of AtTZF5 interacting partners. Surprisingly, most of the AtTZF5 putative interacting proteins are involved in stress responses (Fig 1B and S3 Table). This might be due to the low abundance or absence of the cDNAs that encode proteins involved in mRNA metabolism in the Y-2-H libraries used for screening. Nevertheless, it is not a total surprise to have obtained these results either, because there are precedencies for such protein-protein interactions. For example, cotton TZF protein, GhZFP1 interacts with Responsive to Dehydration 21A (GZIRD21A) protein that is highly conserved with Arabidopsis RD21A [32]. Maldonado-Bonilla et al (2014) reported that AtTZF9 was able to interact with Pathogen-Associated Molecular Pattern (PAMP) activated MPK3 and MPK6 in both cytoplasm and nucleus. In fact, hTTP can also interact with stress related proteins, such as heat shock protein 70 (Hsp70) via its TZF domain [24]. Together, these results suggest a novel mechanism by which plant RR-TZFs participate in stress responses via direct protein-protein interaction, rather than just controlling the stabilities of mRNAs encoding stress response regulators as found in animals (Brook and Blackshear, 2013).

Among 19 stress related proteins, MARD1 and RD21A were selected for in-depth characterization of protein-protein interaction. MARD1 is a zinc finger protein with a zinc finger domain of C-X_2_-C-X_19_-C-X_3_-C at the C-terminus. The N-terminus of MARD1 (residues 1–100) is a proline-rich region, which could be involved in protein-protein interaction. *MARD1* is highly expressed in mature seeds and is induced by ABA. Similar to KO mutants of AtTZF4, 5, and 6 [16], MARD1 KO mutants showed early and phytochrome-independent seed germination. The MARD1 KO mutants were also insensitive to ABA-mediated inhibition of seed germination [42]. On the other hand, RD21A is a papain-like cysteine protease [45–47] involved in abiotic and biotic stress responses [45, 48]. KO mutants of RD21A were hyper-susceptible to the necrotrophic fungal pathogen *Botrytis cinerea* infection [45]. Transcripts of *RD21A* were also strongly induced by abiotic stresses such as high salt [48]. Future genetic analysis (e.g., double mutants) should provide a clue for the roles of MARD1 and RD21A in AtTZF4, 5, and 6- mediated control of seed germination.

In Y-2-H analyses, while MARD1 and RD21A could interact with full-length AtTZF5, they could not interact with TZF and RR-TZF fragments. This is inconsistent with the results of BiFC analysis, where both RR-TZF and TZF fragments could interact with MARD1 and RD21A, respectively. Likewise, both TZF and RR-TZF were able to interact with MARD1 in co-IP analysis. While these results are intriguing, they are in agreement with the findings by Guo et al. (2009) where RR-TZF region is important for cotton TZF (GhZFP1) to interact with cotton RD21A. Although MARD1 and RD21A could interact with AtTZF4, 5, and 6, they could not interact with non-seed-specific AtTZF1 in Y-2-H analysis. This is unlikely due to the instability of AtTZF1 because Western blot analysis could detect a significant level of AtTZF1 protein expression in yeast cells (Fig 4). Arguably, because TZF motif alone is sufficient for protein-protein interaction, AtTZF1 should interact with MARD1 and RD21A in yeast cells due to extremely high conservation in TZF motif [8]. However, it is possible that AtTZF1 might contain a negative interaction domain that prevents its interaction with MARD1 and RD21A. For example, c-Myeloblastosis protein (c-Myb) has a negative domain in the C-terminus that folds back onto the N-terminal DNA binding domain (DBD) upon phosphorylation, thereby impeding its interaction with cofactor proteins [49].

Unlike mammalian TZFs, AtTZF4, 5, and 6 could not interact with decapping complex components including DCP1, 2, and 5 (S2 Fig) in Y-2-H analyses. This might be due to the low conservation of N-terminal sequences between hTTP and AtTZF4, 5, and 6, given their identities are only 25, 33 and 40%, respectively. On the other hand, these interactions might require post-translational modifications such as phosphorylation that do not or inappropriately occur in yeast cells. For example, AtTZF9 is a phosphor-target of MPK3 and MPK6 [33] and DCP1 is also a phosphor-target of MPK6 [50]. Therefore, it is plausible to speculate that phosphorylation of AtTZF4, 5, and 6 may be required for the interaction between AtTZFs and decapping components. Nevertheless, it is actually unclear if hTTP can directly interact with the decapping components, although they are present in the same protein complex in a co-IP analysis [19]. hTTP can also interact with PABPC1 (cytoplasmic PABP) [24] and PABPN1 (Nuclear PABP) [26] via its TZF domain. However, AtTZF4, 5, and 6 did not interact with PABP8 (S2 Fig). This may be due to the difference in interacting domain structure between the two PABPs or between hTTP and AtTZFs. For instance, although TZF domain is conserved between AtTZFs and hTTP, their CCCH spacing patterns are quite different [8]. Even though it has been shown that N and C termini of Arabidopsis PABP8 are conserved with that of human PABPC1 [51], the identities for C and N termini are limited to 66% and 48%, respectively. On the other hand, it is not known what region of human PABPC1 is important for the interaction with hTTP; therefore, it is not possible to draw any comparisons. It is also possible that AtTZF4, 5, and 6 might interact with other PABPs out of the 8 conserved Arabidopsis PABPs [51] that have not been tested in our study.

Despite the negative interactions between AtTZF4/5/6 and DCPs/PABP8, AtTZF4/5/6 could interact with MARD1 and RD21A in both Y-2-H and BiFC analyses. Furthermore, MARD1 and RD21A could co-localize with AtTZF5 and PB (DCP2)/SG (UBP1b) components, respectively (Fig 8). Intriguingly, MARD1/RD21A could co-localize 100% with both DCP2 and UBP1b (Fig 8). In previous reports, it was also observed that PB and SG dual-localized proteins could co-localize 100% with either PB or SG markers when overexpressed in tandem [8, 16]. This could be a common artifact due to unusually high levels of co-expressed proteins in the cells [52]. Perhaps an ideal experiment would be to express the protein of interest (POI) plus both PB and SG marker under native promoter and then determine how POI co-localizes with PB and SG markers simultaneously. Conversely, the BiFC could be combined with co-localization analysis by co-expressing 3 or 4 constructs together (POI#1-CYFP, POI#2-NYFP, plus PB and/or SG marker). However, it would be challenging, if not impossible, to express all 3 (or 4) proteins in equilibrium, and yet the results could still be biased due to the dynamic external and internal control of PB and SG assembly [9]. It is also intriguing that both PBs and SGs could be observed in protoplasts without specific treatments. It is well documented that PB/SG assembly is induced by specific cues and orchestrated by specific signal transduction pathways [9, 44, 52]. However, plant RR-TZFs can often be localized to PBs/SGs when transiently expressed in protoplasts. This is evidenced by a clear PB/SG localization pattern of AtTZF1 [8], AtTZF4-6 [16], AtTZF9 [33], and OsTZF1 [53]. The factors serving as triggers for PB/SG assembly in protoplast transient expression systems have yet to be determined. One possibility is that the high-concentration osmotic reagents, such as mannitol, sorbitol, or salt solutions used in maintaining protoplast turgor pressures, could have served as signals in inducing PB/SG assembly.

In conclusion, we have found that plant TZF proteins preferentially interact with stress responsive proteins. AtTZF4, 5, and 6 can interact with MARD1 and RD21A, and the interactions are likely taken place in PBs and SGs. Future work should focus on the dissection of protein-protein interaction in space and time and under various stimuli, unraveling the roles of AtTZF-MARD1 and AtTZF-RD21A interaction in plant growth and stress responses as well as in post-transcriptional regulation of gene expression, and addressing the molecular mechanisms by which RR-TZF motif use to interact with both RNAs and proteins.

## Supporting Information

S1 Fig Subcellular localization of AtTZF5 and its interacting protein partners. (A) AtTZF5, (B) MARD1, (C) RD21A, (D) PP2A-4, (E) DIN10 and (F) Oleosin. Bar = 10 μm.
(TIFF)

S2 Fig AtTZF4/5/6 do not interact with decapping components (DCP1, 2, and 5) and poly-A binding protein PABP8 in Y-2-H analysis. AtTZF4, 5, and 6 were fused with GAL4 DNA binding domain (BD), whereas DCPs and PABP8 were fused with GAL4 activation domain (AD). Combination of bZIP1 and bZIP25 was used as a positive control.
(TIFF)

S1 Table Oligo primers used for yeast two-hybrid, bimolecular fluorescence complementation, and co-immunoprecipitation assays. (DOC)

S2 Table Potential interacting partners of AtTZF5. (DOC)

S3 Table Putative interacting partners of AtTZF5 are involved in different types of stress responses. (DOC)

S4 Table Tissue expression patterns of putative interacting partners of AtTZF5. (DOC)

S5 Table Summary for protein-protein interaction analyses. (DOC)
