# Supplementary figures and images for: Plant Tandem CCCH Zinc Finger Proteins Interact with ABA, Drought, and Stress Response Regulators in Processing-Bodies and Stress Granules
Source: PLoS One. 2016 Mar 15;11(3):e0151574. doi: 10.1371/journal.pone.0151574 (PMC4792416; doi:10.1371/journal.pone.0151574)

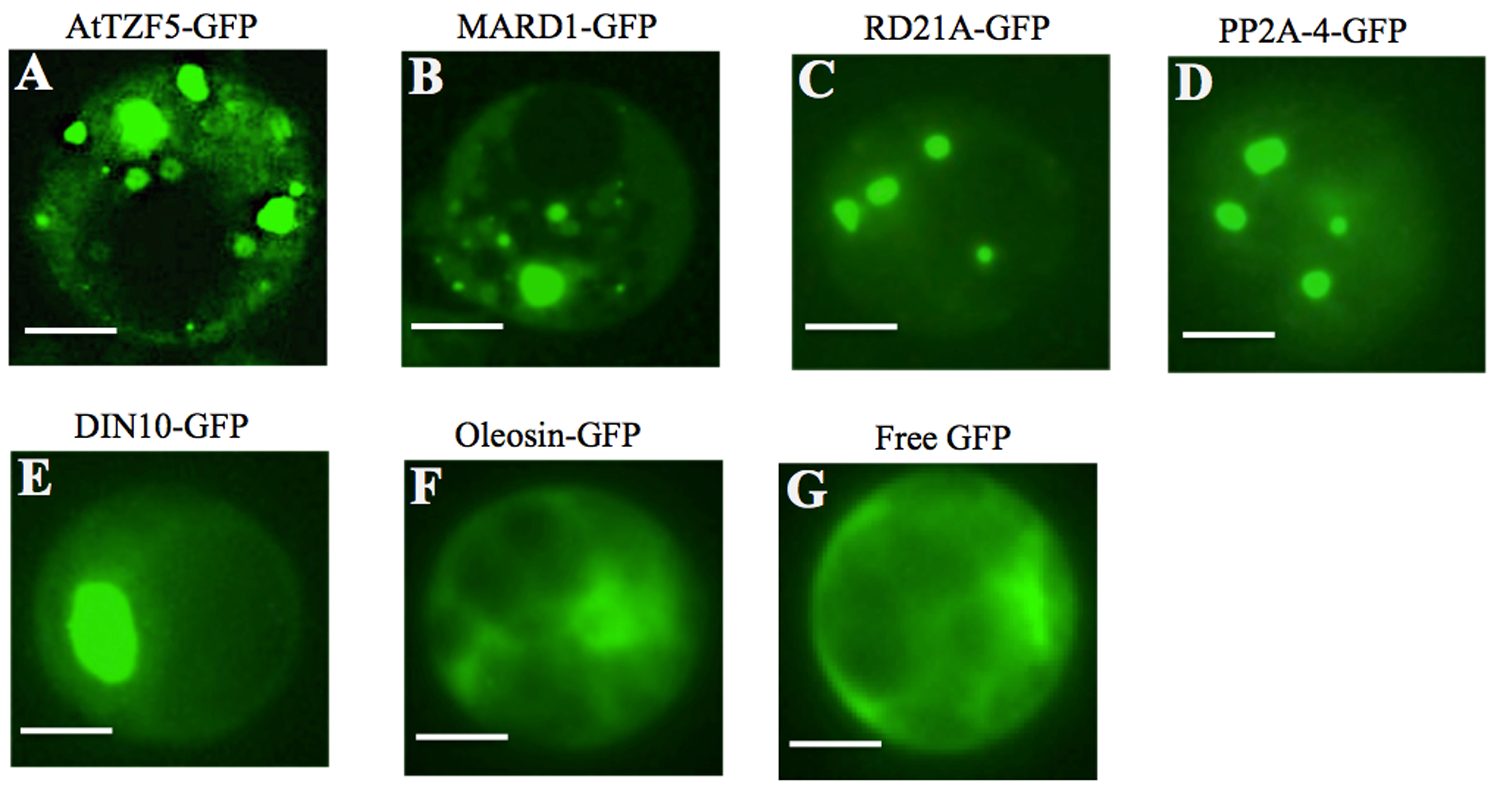

Supplement: S1 Fig — (A) AtTZF5, (B) MARD1, (C) RD21A, (D) PP2A-4, (E) DIN10 and (F) Oleosin. Bar = 10 μm. (TIFF) [file pone.0151574.s001.tiff]

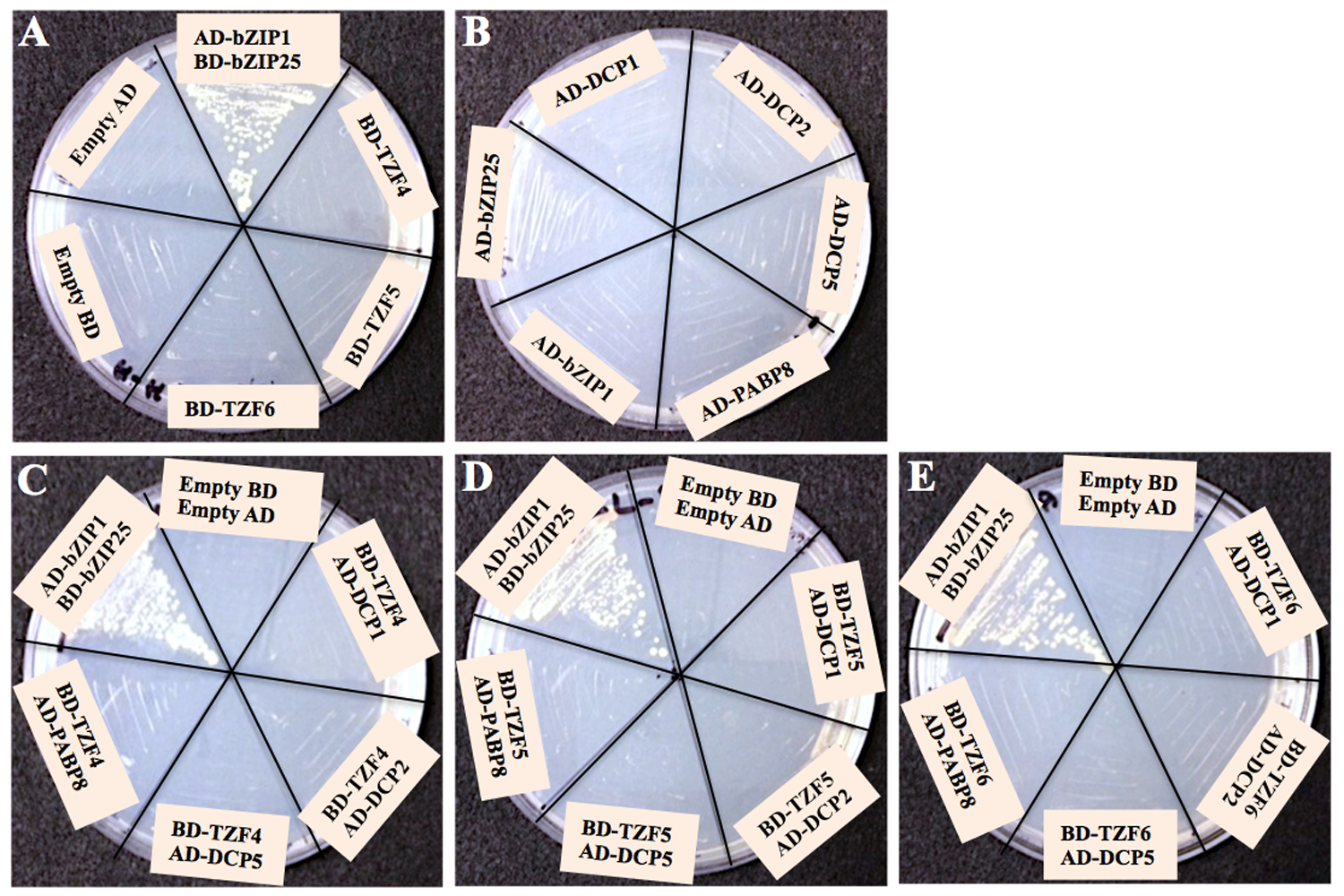

Supplement: S2 Fig — AtTZF4, 5, and 6 were fused with GAL4 DNA binding domain (BD), whereas DCPs and PABP8 were fused with GAL4 activation domain (AD). Combination of bZIP1 and bZIP25 was used as a positive control. (TIFF) [file pone.0151574.s002.tiff]
