# Supplementary material for: Plant Tandem CCCH Zinc Finger Proteins Interact with ABA, Drought, and Stress Response Regulators in Processing-Bodies and Stress Granules
Source: PLoS One. 2016 Mar 15;11(3):e0151574. doi: 10.1371/journal.pone.0151574 (PMC4792416; doi:10.1371/journal.pone.0151574)
Supplement: S1 Table — (DOC) [file pone.0151574.s003.doc]

**S1 Table**. Oligo primers used for yeast two-hybrid, bimolecular fluorescence complementation, and co-immunoprecipitation assays

| **Yeast two-hybrid (Y-2-H) assays** | |
| --- | --- |
| *AtTZF1* (At2g25900) | 5’-GGAATTCCATATGATGATGATGATCGGCGAAAATAAAAA-3’  5’- ACGCGTCGACTCAACCGAGTGAGTTCTCTCT-3’ |
| *AtTZF4*  (At1g03790) | 5’-GGAATTCCATATGATGGCGTCCTTCAAGTTGATG-3’  5’-ACGCGTCGACTCACCATTCCAAGGAATGGCT-3’ |
| *AtTZF5*  (At5g44260) | 5’-GGAATTCCATATGATGGACGTCGAACATCACAAA-3’  5’-ACGCGTCGAC TCATGTCAAAAGATCGTTCACC-3’ |
| *AtTZF6*  (At5g07500) | 5’-GGAATTCCATATGATGTTGAAAAGTGCAAGTCCAA-3’  5’-ACGCGTCGACTTAATCGACCAACTCTGAGATC-3’ |
| AtTZF5-RR-TZF | 5’-GGAATTCCATATGATGTTCTCCTCCGACGAGTTTCGC-3’  5’-ACGCGTCGACAGGTAATACACGTAACTGCTC-3’ |
| AtTZF5-TZF | 5’-GGAATTCCATATGATGCATCCCGGAGAAAAAGCTCG -3’  5’- ACGCGTCGACAGGTAATACACGTAACTGCTC-3’ |
| *AtMARD1*  (At3g63210) | 5’- CCGGAATTCATGCTTAGAAACAAACCTAGAG-3’  5’- CCGCTCGAGCCTAGGTCTCCATTTGATCAAG-3’ |
| *RD21A* (At1g47128) | 5’- CCGGAATTCATGGGGTTCCTTAAGCCAAC-3’  5’- CTAGTCTAGACTTAGGCAATGTTCTTTCTGCC-3’ |
| **Bimolecular fluorescence complementation (BiFC)** | |
| *AtTZF4*  (At1g03790) | 5’-CCGCTCGAGGATGGATGTCGTTTGTACGGAA-3’ 5’CGCGGATCCAGTCAAGAGATCATTGACCC-3’ |
| *AtTZF5*  (At5g44260) | 5’-CCGCTCGAGGATGGACGTCGAACATCACAAATCC-3’  5’- CTAGACTAGTTGTCAAAAGATCGTTCACCC-3’ |
| *AtTZF6*  (At5g07500) | 5’- CCGCTCGAGGATGTTGAAAAGTGCAAGTCCAA-3’  5’-CGCGGATCCATCGACCAACTCTGAGATC-3’ |
| TZF5-RR-TZF | 5’- CCGCTCGAGGATGTACGCTGGAGACCATTTCCGG-3’  5’-CGCGGATCCTGGAAGAACTCTCAGCTGACG-3’ |
| TZF5-TZF | 5’- CCGCTCGAGGATGCATCCCGGTGAAAAGGCTC-3’  5’- CGCGGATCCTGGAAGAACTCTCAGCTGACG-3’ |
| *AtMARD1*  (At3g63210) | 5’- ACGCGTCGACATGCTTAGAAACAAACCTAGAG-3’  5’- CGCGGATCCGGTCTCCATTTGATCAAGAAGCA-3’ |
| *AtRD21A*  (At1g47128) | 5’- CCGCTCGAGCATGGGGTTCCTTAAGCCAAC-3’  5’- CGCGGATCCGGCAATGTTCTTTCTGCCTTG-3’ |
| **Co-Immunoprecipitation assay** | |
| *AtTZF5* (At5g44260) | 5’-CGGGGTACCATGGACGTCGAACATCACAA-3’  5’-ATAAGAATGCGGCCGCAATGTCAAAAGATCGTTCACCC-3’ |
| TZF5-RR-TZF | 5’-CACCATGTACGCTGGAGACCATTTCCGG-3’  5’-TGGAAGAACTCTCAGCTGACG-3’ |
| TZF5-TZF | 5’-CACCATGCATCCCGGTGAAAAGGCTC-3’  5’-TGGAAGAACTCTCAGCTGACG-3’ |
