# Supplementary material for: Plant Tandem CCCH Zinc Finger Proteins Interact with ABA, Drought, and Stress Response Regulators in Processing-Bodies and Stress Granules
Source: PLoS One. 2016 Mar 15;11(3):e0151574. doi: 10.1371/journal.pone.0151574 (PMC4792416; doi:10.1371/journal.pone.0151574)
Supplement: S2 Table — (DOC) [file pone.0151574.s004.doc]

**S2 Table.** Potential interacting partners of AtTZF5

| Colony No. | AGI | Protein | No. of hits |
| --- | --- | --- | --- |
| 3.1 | At5g20250 | DIN10 | 1 |
| 5.1, 60.1 | At4g26970 | ACONITASE 2 | 2 |
| 6.2 | At1g78040 | Pollen ole e 1 | 1 |
| 15.1, 37.1 | At4g24760 | Alpha-beta-hydrolases | 2 |
| 16.2, 38.1 | At5g56100 | Glycine rich protein -Oleosin | 3 |
| 18.1 | At3g63210 | MARD1 | 1 |
| 21.1, 40.1, 65.1, 70.3 | At4g35090 | CAT2 | 4 |
| 22.1 | At4g25340 | FKBP53 | 1 |
| 23.1, 23.4, 32.2 | At2g29630 | THIC | 3 |
| 23.1, 52.1 | At3g62410 | CP12-2 | 2 |
| 24.1, 53.1 | At5g32107 | Transposable element gene | 2 |
| 25.1, 30.2 | At5g42620 | Metalloendopeptidases | 2 |
| 29.1, 29.4 | At3g58500 | PP2A-4 | 3 |
| 31.2 | At5g04540 | MTM2 | 1 |
| 41.1 | At4g30860 | ASH1 related 3 | 1 |
| 42.1 | At3g16420 | PBP1 | 1 |
| 43.1 | At5g03360 | DC1 domain-containing protein | 1 |
| 44.10 | At5g19660 | Site 1 protease | 1 |
| 45.4 | At1g20260 | VAB3 | 1 |
| 46.1 | At3g09260 | PYK10, PSR3.1, LEB | 1 |
| 47.1 | At3g05545 | RING/U box family protein | 1 |
| 51.10 | At1g52200 | PLAC8 family protein | 1 |
| 55.10 | At3g48680 | ATCAL2 | 1 |
| 58.1 | At3g49055 | Unknown protein | 1 |
| 59.1 | At1g47128 | RD21A | 1 |
| 60.1 | At4g26970 | ACONITASE 2 | 2 |
| 61.1 | At4g13130 | Zinc ion binding protein | 1 |
| 62.1 | At2g34430 | LHbB1, LHCB1 | 1 |
| 63.1 | At1g64980 | CDI | 1 |
| 64.2 | At5g27280 | Zim-17 type zinc finger protein | 1 |
| 67.1 | At2g20890 | Photosystem II PSB29 protein | 1 |
| 68.2 | At1g57700 | Protein kinase super family protein | 1 |
| 72.3 | AtCG01090 | NDH1 | 1 |
| 73.3 | At5g35630 | GS2, GLN2, ATGSL1 | 1 |
| 74.1 | At5g12150 | RHO GTPase activation protein | 1 |
| 77.2 | At1g48630 | RACK1B | 1 |
| 78.1 | At5g60360 | AALP, ALP, SAG 2 | 1 |
