# Supplementary material for: Plant Tandem CCCH Zinc Finger Proteins Interact with ABA, Drought, and Stress Response Regulators in Processing-Bodies and Stress Granules
Source: PLoS One. 2016 Mar 15;11(3):e0151574. doi: 10.1371/journal.pone.0151574 (PMC4792416; doi:10.1371/journal.pone.0151574)
Supplement: S3 Table — (DOC) [file pone.0151574.s005.doc]

**S3 Table.** Putative interacting partners of AtTZF5 are involved in different types of stress responses

| AGI No. | Salt | Oxidative | Osmotic | Cold | Heat | ABA Stimulus | Pathogenesis related | Hypoxia |
| --- | --- | --- | --- | --- | --- | --- | --- | --- |
| At1g47128 | ✔ |  |  |  | ✔ |  |  |  |
| At3g48680 | ✔ |  |  |  |  | ✔ |  |  |
| At5g19660 | ✔ |  | ✔ |  |  |  |  |  |
| At5g20250 | ✔ | ✔ |  | ✔ |  |  |  |  |
| At5g35630 | ✔ |  | ✔ | ✔ | ✔ |  |  |  |
| At5g60360 | ✔ |  | ✔ |  | ✔ |  |  |  |
| At3g09260 | ✔ |  | ✔ | ✔ |  |  | ✔ |  |
| At2g29630 |  |  | ✔ |  |  | ✔ |  |  |
| At3g62410 |  |  |  | ✔ | ✔ |  |  | ✔ |
| At5g04540 |  |  | ✔ |  |  |  | ✔ |  |
| At4g35090 |  | ✔ |  | ✔ |  |  |  |  |
| At1g52200 |  | ✔ |  |  |  |  |  |  |
| At4g26970 |  | ✔ |  |  |  |  |  |  |
| At3g16420 |  | ✔ |  |  |  |  |  |  |
| At5g03360 |  | ✔ |  |  |  |  |  |  |
| At4g13130 |  | ✔ |  |  |  |  |  |  |
| At1g48630 |  |  |  |  |  | ✔ |  |  |
| At3g63210 |  |  |  |  |  | ✔ |  |  |
| At1g64980 |  |  |  |  |  |  | ✔ |  |
