# Supplementary material for: Plant Tandem CCCH Zinc Finger Proteins Interact with ABA, Drought, and Stress Response Regulators in Processing-Bodies and Stress Granules
Source: PLoS One. 2016 Mar 15;11(3):e0151574. doi: 10.1371/journal.pone.0151574 (PMC4792416; doi:10.1371/journal.pone.0151574)
Supplement: S4 Table — (DOC) [file pone.0151574.s006.doc]

**S4 Table.** Tissue expression patterns of putative interacting partners of AtTZF5.

| | Seed specific | Expressed in seeds | Expressed in other tissues | | --- | --- | --- | | At1g20260 | At1g78040 | At2g34430 | | At3g05545 | At1g47128 | At4g13130 | | At3g16420 | At1g48630 | At5g12150 | | At3g48680 | At1g52200 | At5g35630 | | At3g58500 | At1g57700 | At5g60360.3 | | At4g26970 | At2g20890 | AtCG01090 | | At5g04540 | At2g29630 |  | | At5g56100 | At3g09260 |  | |  | At3g62410 |  | |  | At3g63210 |  | |  | At4g25340 |  | |  | At4g24760 |  | |  | At4g30860 |  | |  | At4g35090 |  | |  | At5g03360 |  | |  | At5g19660 |  | |  | At5g20250 |  | |  | At5g27280 |  | |  | At1g64980 |  | |  | At5g42620 |  | |
| --- | --- | --- | --- | --- | --- | --- | --- | --- | --- | --- | --- | --- | --- | --- | --- | --- | --- | --- | --- | --- | --- | --- | --- | --- | --- | --- | --- | --- | --- | --- | --- | --- | --- | --- | --- | --- | --- | --- | --- | --- | --- | --- | --- | --- | --- | --- | --- | --- | --- | --- | --- | --- | --- | --- | --- | --- | --- | --- | --- | --- | --- | --- | --- |
