# Supplementary material for: Plant Tandem CCCH Zinc Finger Proteins Interact with ABA, Drought, and Stress Response Regulators in Processing-Bodies and Stress Granules
Source: PLoS One. 2016 Mar 15;11(3):e0151574. doi: 10.1371/journal.pone.0151574 (PMC4792416; doi:10.1371/journal.pone.0151574)
Supplement: S5 Table — (DOC) [file pone.0151574.s007.doc]

|  |  | | |
| --- | --- | --- | --- |
|  |  |  |  |

**S5 Table.** Summary for protein-protein interaction analyses

|  | Yeast two-hybrid | Bimolecular Fluorescence Complement | Coimmunoprecipitation |
| --- | --- | --- | --- |
| AtTZF5 | ✔ | ✔ | ✔ |
| AtTZF5-TZF | ✖ | ✔ | ✔ |
| AtTZF5-RR-TZF | ✖ | ✔ | ✔ |
| AtTZF1 | ✖ | **−** | **−** |
| AtTZF1-TZF | − | − | − |
| AtTZF1-RR-TZF | − | − | − |

✔ = positive results; ✖ = negative results; − = not done
